# A Temperature‐Responsive Smart Europium Metal‐Organic Framework Switch for Reversible Capture and Release of Intrinsic Eu^3+^ Ions

**DOI:** 10.1002/advs.201500012

**Published:** 2015-03-10

**Authors:** Min Zhu, Xue‐Zhi Song, Shu‐Yan Song, Shu‐Na Zhao, Xing Meng, Lan‐Lan Wu, Cheng Wang, Hong‐Jie Zhang

**Affiliations:** ^1^ State Key Laboratory of Rare Earth Resource UtilizationChangchun Institute of Applied ChemistryChinese Academy of Sciences5625 Renmin StreetChangchun130022P. R. China; ^2^ University of Chinese Academy of SciencesBeijing100049P. R. China

**Keywords:** dynamic bonds, explosive detection, host‐guest system, phase transitions, smart frameworks

## Abstract

Stimuli‐responsive structural transformations are emerging as a scaffold to develop a charming class of smart materials. A **EuL** metal‐organic framework (MOF) undergoes a reversible temperature‐stimulated single‐crystal to single‐crystal transformation, showing a specific behavior of fast capture/release of free Eu^3+^ in the channels at low and room temperatures. At room temperature, compound **1a** is obtained with one free carboxylate group severing as further hook, featuring one‐dimensional square channels filled with intrinsic free europium ions. Trigged by lowering the ambient temperature, **1b** is gained. In **1b**, the intrinsic free europium ions can be fast captured by the carboxylate‐hooks anchored in the framework, resulting in the structural change and its channel distortion. To the best of our knowledge, this is the first report of such a rapid and reversible switch stemming from dynamic control between noncovalent and covalent Eu–ligand interactions. Utilizing **EuL** MOF to detect highly explosive 2,4,6‐trinitrophenol at room temperature and low temperature provides a glimpse into the potential of this material in fluorescence sensors.

## 1 Introduction

Flexible crystalline solids are endowed with the ability to change their structures in response to external stimuli, and have recently become appealing to materials scientists.^1^ This so‐called dynamism might be key to developing a charming class of smart materials because their structurally dependent physical, chemical, and mechanic properties could be cooperatively moderated.^2^ For example, single‐crystal devices generally require a crystal to tolerate nondestructive internal changes to perform a specific function.^3^ However, single crystals are usually brittle and tend to crack because of long‐range strain from their disrupted internal periodicity. Single‐crystal to single‐crystal (SCSC) transformations, which could preserve the molecular‐level continuity and retain their single‐crystal character after an atomic solid‐state reorganization, allow the internal changes to be uniformly effected, and the resultant crystals can be readily characterized via single crystal X‐ray diffraction.^4^ In some cases, this characterization could unveil the desired dynamic process for the transformation, whereas normally solid‐state reactions involve amorphous phases and are often more difficult to determine.^5^ SCSC transformations provide a unique approach for directly observing molecular rearrangements, and offer new materials that cannot be achieved under conventional conditions.^6^ Although the SCSC processes occur accidentally, interest in them has increased for chemists working in the field of metal‐organic crystalline materials.

Porous metal‐organic frameworks (MOFs) and their related materials have attracted escalating attention due to their promising applications in chemistry and materials science.^7^ The metal‐centered guests, including kinds of simple, hydrated, solvent‐surrounded and ligand‐complexed, could impart or enrich the MOF materials with various functional properties from luminescence to catalysis.^8^ Particularly, the host–guest interactions between the metal‐relied guests and parent framework usually focus on noncovalent effects, such as electrostatics, H‐bonding and so on. However, recently in the work of Bu and co‐workers, they showed a new mechanism for controlling the structural topology through covalent interactions between the internal metal ions/clusters and hooks originating from the spare functional groups on the polytopic organic building blocks.^9^ Thus, it indicated that metal ions/clusters could be covalently captured by the remaining groups in a concerted manner. This idea likely holds great promise for modifying the structural properties of the framework. However, the captured metal ions/clusters are always tightly fastened in the pores via stable covalent interactions, and cannot handle flexible modifications. In dynamic chemistry, the term “dynamic bond” can be defined as a class of bond that can selectively undergo reversible breaking and reformation upon exposure to certain environmental factors.^10^ Reversible or dynamic covalent chemistry involving such “dynamic bond” has a long history in polymer science, and a wide range of stimuli‐responsive materials with many different mechanisms to “read” and respond to the input stimulus have been exploited.^11^ The use of dynamic covalent chemistry to program a fundamental response has been a new trend in designing adaptive materials. This could be a strategic approach to harvesting MOF‐based smart materials, which require dynamic control between their noncovalent and covalent interactions.

SCSC conversions in MOFs have emerged as an interesting phenomenon for exploring new MOF materials and enhancing their functions.^12^ Most reported SCSC transitions involve solvation and desolvation, ligands and solvent exchanges as well as cation exchanges in the MOFs, whereas structural conversions related to metal guest–ligand interactions are rarely reported. As an extension of our work on lanthanide MOF materials,^13^ a new three‐dimensional (3D) **EuL** MOF (**1a**) entrapping disordered Eu^3+^ was obtained. It can quickly capture and release Eu^3+^ in its channels at low and room temperatures, respectively, through SCSC transformations. To the best of our knowledge, this is the first report of such a rapid and reversible switch stemming from dynamic Eu‐ligand bonds.

## 2 Results and Discussion

### 2.1 Structures of Two Compounds

**Figure 1:**
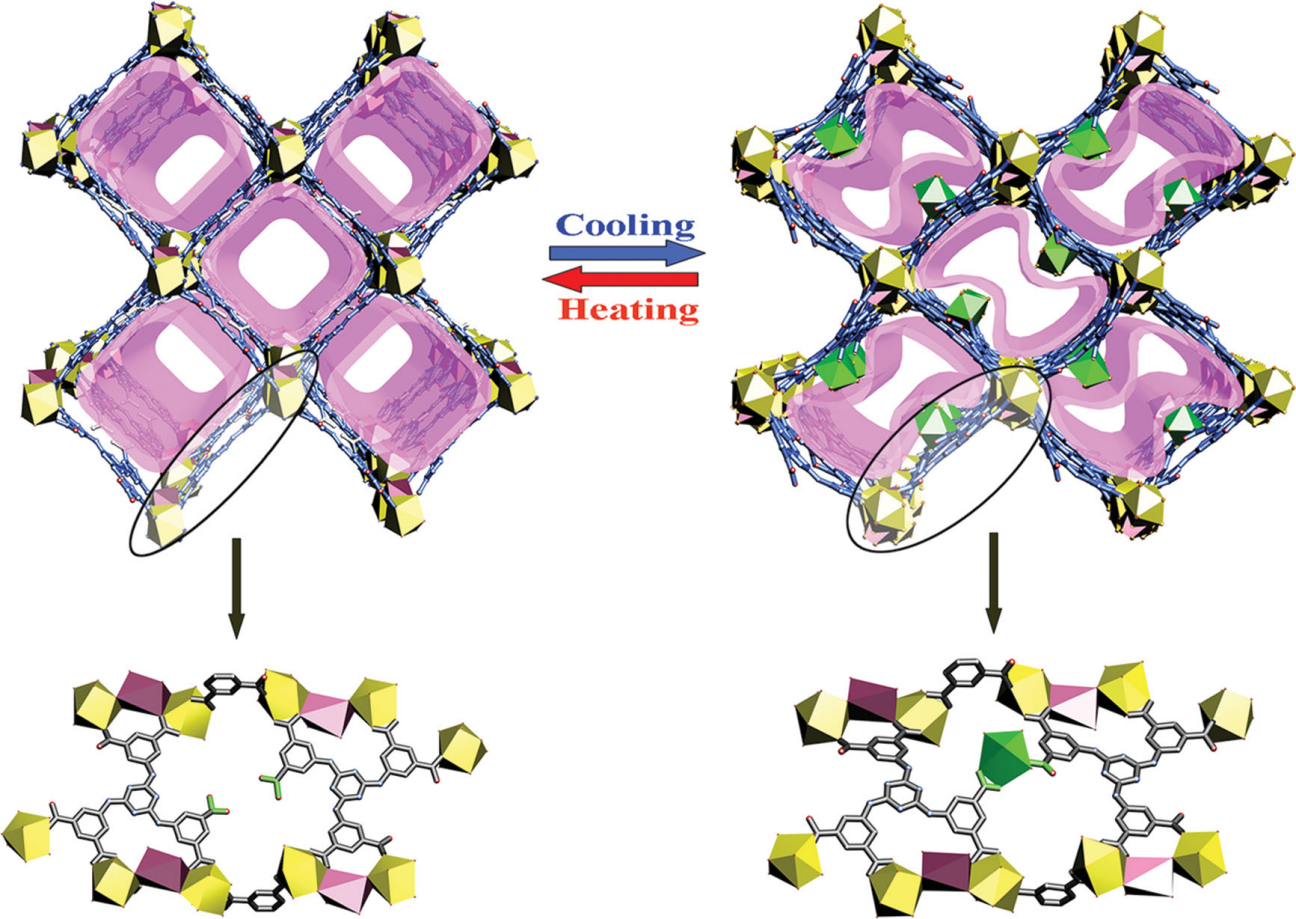
SCSC transformations of the porous framework between **1a** (left) and **1b** (right) induced by the temperature. Top: the overall view of the 3D structures along the *c* axis. Bottom: the local enlarged view of the structural difference.

X‐ray analysis at 293 K reveals **EuL** MOF (**1a**) crystallizes in the monoclinic space group *C*2/*c*. The framework is constructed by one Eu^3+^, one‐half Na^+^, one **L** molecule, and one coordinated DMF molecule. The Eu^3+^ center is nine‐coordinated by eight oxygen atoms from five carboxyl groups in **L** and one oxygen atom (O13) from incomplete DMF in a tricapped trigonal prism coordination geometry (Figure S1a, Supporting Information). The Eu–O distances range from 2.381(7) to 2.564(6) Å. The Na^+^ is six‐coordinated by four oxygen atoms from four carboxyl groups in **L** and two aqua ligands, connecting adjacent Eu^3+^ to Eu_2_Na units through the **L** bridging carboxyl group (Figure S2a, Supporting Information). The Na–O distances range from 2.387(6) to 2.846(7) Å. The ligand shows a slightly distorted *exo*‐conformation with two side benzene rings (called *a* and *b*) up and one (called *c*) down relative to the bridging triazine core. The dihedral angles between the side benzene rings (*a*, *b,* and *c*) and the central triazine ring are 9.216°, 6.316°, and 8.439°, respectively. The distances between the side rings' centroid and the central ring's plane are 0.421(3), 0.251(1), and 0.246(8) Å. Three carboxyl groups adopt the chelated mode and two adopt the monodentate fashion to coordinate with five Eu^3+^, the last carboxyl group was left and uncoordinated (Figure S5a, Supporting Information). The overall framework built from Eu_2_Na units and **L** ligands is a 3D porous framework that has a one‐dimensional (1D) large square channel along the *c* axis (**Figure** **1**a). The Eu_2_Na units behave as the corner and the ligands as the edge. Considering the van der Waals radius, the channel dimension is approximately 14.038(9) × 14.038(9) Å (calculated from the shortest Eu…Eu distance). The distances between two adjacent uncoordinated carboxyl groups are 4.825(7) and 4.878(3) Å (corresponding to opposite oxygen atoms).

**Figure 2:**
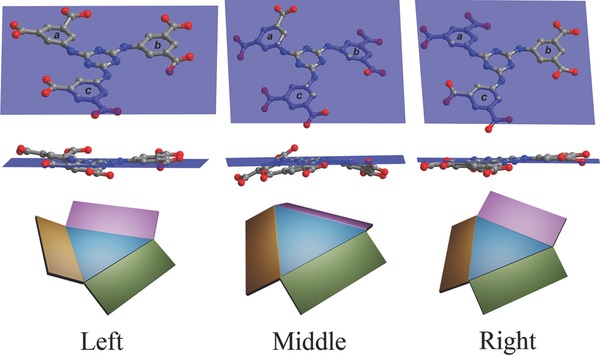
View of the different conformations of **L** in compounds **1a** and **1b**. Top‐to‐down: top view, side view, and schematic view. Left‐to‐right: **L** in **1a** (**L1**), two different **L** anions in **1b** (**L2** and **L3**). Color codes (bottom): blue (triazine core), brown (ring a), pink (ring b), and green (ring c).

We successfully collected the single‐crystal diffraction data at 193 K, and found that the space group of **EuL** transformed from *C*2/*c* to *P*2_1_/*n*, making a new **EuL** MOF (**1b**), with no apparent difference between **1a** and **1b** in color, shape, and size of the crystals. There are three crystallographically independent Eu centers in **1b**. Eu1 and Eu2 remain the coordination fashion of Eu1 in **1a** and Eu3 is eight‐coordinated by three oxygen atoms from two carboxyl groups in **L**, three oxygen atoms from three DMF molecules and two aqua ligands, furnishing a trigonal dodecahedron geometry (Figure S1b, Supporting Information). The Eu_2_Na units in **1b** are almost identical to those in **1a** (Figure S2b, Supporting Information). Compared with the **L** in **1a**, the ligands exhibit two conformations due to their benzene rings rotations, and the spare carboxyl groups adopt chelated and monodentate coordination with Eu3 (Figure S5b, Supporting Information). For the chelated **L**, the three side rings all rotate around the bridging C–N bonds down from the triazine core in an *endo*‐conformation, with dihedral angles of 23.891°, 17.781°, and 12.055° for *a*, *b,* and *c*, respectively, which is a more severe distortion than that observed for **1a** (**Figure** **2**, middle). The distances between the side rings' centroid and the central ring's plane are 0.464(4), 0.715(3), and 0.463(1) Å. The monodentate **L** shows a slight distortion, with two side rings angled down and one angled up from the central ring in an *exo*‐conformation (Figure 2, right). The dihedral angles are 8.044°, 3.929°, and 7.579°, and the corresponding distances are 0.457(3), 0.259(5), and 0.230(5) Å, respectively. The overall framework of **1b** is nearly identical to that of **1a**, however, the most significant structural change is the additional Eu3 ions in the pores coordinated to the **L** in the framework, which distort the entire framework (Figure 1b). The dimension of the elliptical‐like distorted channels containing Eu3 in **1b** changes to 9.056(2) × 16.096(3) Å (corresponding to the Eu3…Eu3 distance at the pore). In compound **1a**, each **L** anion connects three Eu_2_Na secondary building units (SBUs), thus the **L** anion can be defined as a 3‐connected node. The inorganic trinuclear Eu_2_Na SBU can be reduced to a 6‐connected node, linking six **L** anions. On the basis of this simplification, compound **1a** possesses a rare binodal (3,6)‐connected **ant**‐type topology, with the Schläfli symbol of (4^2^·6)_2_(4^4^·6^2^·8^8^·10). When the additional Eu3 ion was captured, **L** anion connects each other through the Eu3 center to serve as a 4‐connected node. Thus, the resultant framework of **1b** is a binodal (4,6)‐connected new topology with the Schläfli symbol of (4^2^·5^2^·6^2^)_2_(4^4^·5^7^·6^2^·7^2^), as shown in Figure S6, Supporting Information.

### 2.2 Single‐Crystal to Single‐Crystal Transformation Process and Mechanism

**Figure 3:**
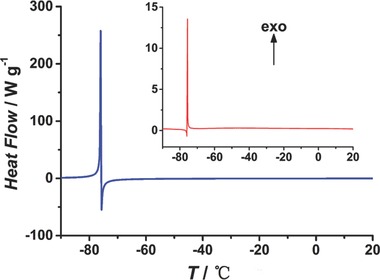
Low temperature DSC analyses of **EuL** from 20 to −90 °C (blue line), and from −90 to 20 °C (red line).

To clarify that the above structural conversion upon exposure to room and low temperatures occurs in a SCSC manner, monitoring the single‐crystal diffractions of the same single crystal in the low temperature region is thus highly desirable. Keeping the crystal at −80 °C within approximately 10 min yielded the cell parameter for **1b**. This result indicated the SCSC transformation from **1a** to **1b** is rapid. The same single‐crystal was kept at room temperature for approximately 30 min, and the cell parameter for **1a** was collected again, which confirmed that the SCSC process is fast and reversible (Scheme S1, Supporting Information). Although similar low temperature‐induced SCSC transformations have been previously reported in MOFs,^14^ they were slow or irreversible, whereas a fast and reversible SCSC conversion has not been reported to date. To more clearly observe the reversibility, the diffractions of the same single crystal from −80 °C back to room temperature were monitored. The cell parameters for **1b** gradually changed to that of **1a** (**Table** **1**). A temperature‐down DSC analysis from 20 °C to −90 °C showed a pair of narrow, sharp peaks at about −75.8 °C, which demonstrates the fast phase transformation for **EuL**. An endothermic peak followed by an exothermic one immediately without any plateau separation indicates a drastic two‐step phase transition with a transient intermediate state. The reversibility of this special phase transformation was verified by a similar profile of DSC curve with the temperature increased from −90 °C to 20 °C (**Figure** **3**). The simulated PXRD patterns for **1a** and **1b** are quite different (Figure S9, Supporting Information). Additionally, the **EuL** compounds were further characterized by thermogravimetric analysis and Fourier transform infrared spectrum (Figures S7 and S8, Supporting Information).

**Table 1:** The cell parameters of the same single‐crystal with the temperature gradually increased from 193 to 293 K

Temperature	193 K	203 K	223 K	248 K	293 K
Crystal system	Monoclinic P	Monoclinic P	Monoclinic P	Monoclinic P	Monoclinic C
*a* [Å]	26.06	26.11	26.08	26.10	23.80
*b* [Å]	21.63	21.73	21.90	22.13	24.53
*c* [Å]	31.25	31.27	31.30	31.32	31.20
*β* [º]	106.75	106.81	107.04	107.21	105.86
*V* [Å^3^]	16 869	16 984	17 095	17 281	17 519

**Figure 4:**
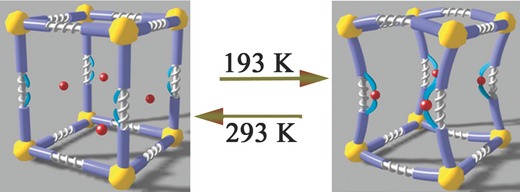
Schematic mechanism for the SCSC transformation. An additional detailed video is included in the Supporting Information.

To further demonstrate the transformation mechanism, we compared the structures of **1a** and **1b**. Three Eu^3+^ were found in framework **1b**, but only two were found in framework **1a** (taking the symmetry into consideration). Because the number of Eu^3+^ ions in the structure is constant before and after the transformation, there must be one additional disordered Eu^3+^ in the channel of **1a**, further confirmed by ICP analysis (Eu/Na ratio = 2.78); however, single‐crystal analysis could not locate the exact position because of its intrinsically high disorder. Thus **1a** can be formulated as (H_3_O^+^)Eu_0.5_[EuNa_0.5_ **L**(DMF)(H_2_O)]·(solvent)*_x_*, with H_3_O^+^ to balance the charge. Keeping the crystal of **1a** at low temperature quickly slows the disordered and rapid Eu^3+^ movement in the pores. When the ion crosses the channel edge, it is rapidly and completely captured by two free carboxyl groups in the framework to form Eu–OOC covalent bonds. This process requires the ligand molecules to simultaneously orient and cooperatively distort in a concerted fashion to suit the capture of Eu^3+^, and act as hooks that form covalent metal–ligand bonds. The ligands conformations changed as the benzene ring rotates around the triazine core, which distort the framework (Figure 2). When the temperature is gradually increased to room temperature, the Eu^3+^ vibration becomes increasingly violent, the captured Eu^3+^ finally escapes the hooks into the channels and the structure is regained (**Figure** **4**). As documented, the Ln–ligand bond has ever shown the dynamics to some extent, and could be reversibly rearranged under pressure or broken and reformed by heating.^15^ This case is intriguing because the metal cation guests could be controlled, captured, and released through smart Eu–L interactions in a SCSC manner. The synergistic effects of the metal guests, carboxyl hooks, and dynamic interactions contribute to the SCSC processes. After the transformation, **1b** could be formulated as (H_3_O^+^)_2_[Eu_3_Na**L**_2_(DMF)_5_(H_2_O)_2_].(solvent)*_y_*.

### 2.3 Photoluminescent Properties and Sensing of the Highly Explosive TNP

The Eu^3+^ ions as nodes and luminescent center, the **L** ligands as linkers and antenna in the framework could impart intriguing luminescent properties. The excitation spectrum of **1a**, monitored under the characteristic emission (617 nm) of the Eu^3+^ ion, exhibits a broad band from 250 to 370 nm with a maximum at 345 nm, and a series of sharp lines. The broad excitation band is assigned to the absorption of the organic ligands, while the sharp line at 395 nm and 465 nm could be assigned to ^7^F_0_→^5^L_6_ and ^7^F_0_→^5^D_2_ transitions, respectively. The emission spectrum of **1a**, upon excitation at 345 nm, reveals well‐resolved magnified luminescence of the f‐f transitions, attributed to the energy transfer from **L** ligands to Eu^3+^ ions. It shows five major peaks centered at 579, 592, 617, 653, and 700 nm, corresponding to the f‐f electronic transitions (^5^D_0_→^7^F_J_, *J* = 0–4) of the Eu^3+^ ion, with the hypersensitive ^5^D_0_→^7^F_2_ transition dominating the spectrum (Figure S10, Supporting Information). Furthermore, the high intensity ratio ^5^D_0_→^7^F_2_/^5^D_0_→^7^F_1_ and the presence of the weak symmetry forbidden ^5^D_0_→^7^F_0_ emission suggests that the Eu^3+^ ions occupy low‐symmetry coordination sites with no inversion centers, consistent with the result of X‐ray structural analysis.^16^,[[qv: 13d]] By decreasing the temperature from 300 to 10 K, the luminescent intensity of the **EuL** MOF material gradually increase because that the nonradiative deactivation process arising from the high‐energy OH, NH, and CH oscillations could be minimized at low temperature (Figure S11, Supporting Information).^17^ It is further confirmed by a gradual increase fluorescence lifetime of ^5^D_0_ decay from 0.8294 ms at 300 K to 0.9267 ms at 10 K (Table S2, Supporting Information).

**Figure 5:**
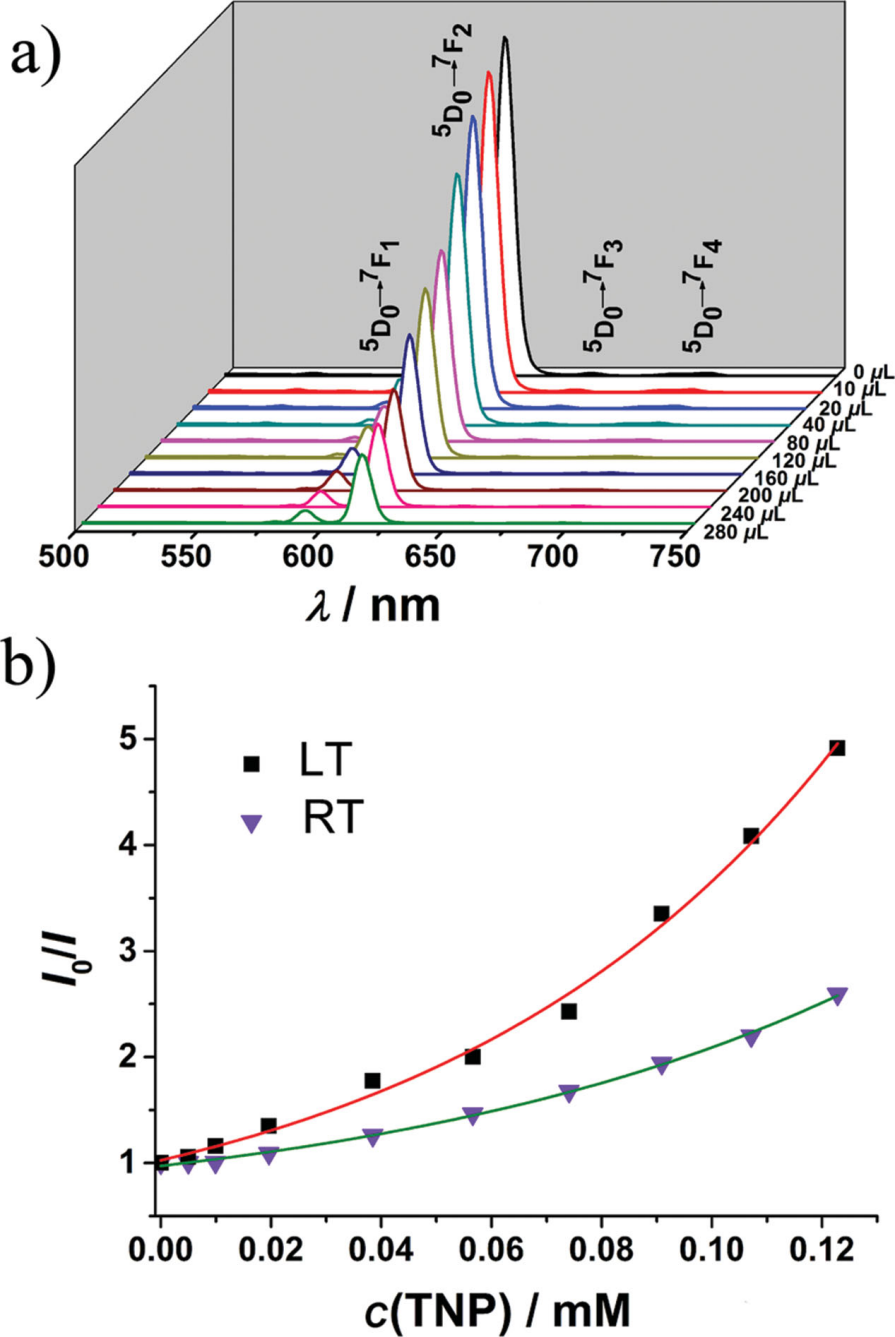
a) The quenching effects on TNP at low temperature with increasing TNP concentration. b) Stern–Volmer plots of *I*_0_/*I* versus the TNP concentration at room and low temperatures.

The 1D channel in our designed **EuL** MOF with bright‐red luminescence may facilitate the preconcentration and recognition of a targeted analyte. The detection process for electron‐deficient nitro‐compounds is mainly based on fluorescence quenching via donor–acceptor electron transfer mechanism. Under room temperature, 2,4,6‐trinitrophenol (TNP), 4‐nitrophenol (4‐NP), 3‐nitrophenol (3‐NP), *ortho*‐nitrotoluene (*o*‐NT), *meta*‐nitrotoluene (*m*‐NT), nitrobenzene (NB), and nitromethane (NM) can weaken the photoluminescent intensity of the **1a** emulsion to different degrees with the quenching efficiency order of TNP > 4‐NP > 3‐NP >*m*‐NT >*o*‐NT > NB > NM (Figure S13, Supporting Information). The highest quenching percentage (QP) of 61.42% for TNP comparing with the other nitro‐compounds at room temperature indicated a high selectivity for TNP. It highlights our material as an excellent candidate for detecting highly explosive TNP with superior selectivity and sensitivity. The presence of two crystallographic phases inspired us to investigate the sensing behaviors towards TNP at room temperature (RT) and low temperature (LT). The fluorescence quenching titration experiments demonstrated that the luminescent intensity decreased continuously upon incremental addition of TNP in THF (1 × 10^−3^ m), with a detectable emissive response at low concentration (4.98 × 10^−6^ m, **Figure** **5**a and Figure S14, Supporting Information). To further quantify the quenching efficiency, the Stern–Volmer plots of the relative luminescent intensity (*I*_0_/*I*) versus the TNP concentration are shown in Figure 5b, where *I*_0_ and *I* are the fluorescence intensity of the emulsion in the absence and presence of the analyte, respectively. Nonlinear Stern–Volmer curves (RT and LT) can be well‐fitted by the exponential equations of *I*_0_/*I* = 0.52e^11446.85^ [TNP] + 0.45 and *I*_0_/*I* = 0.89e^13776.95^[TNP] + 0.14 with quenching constants of 5.97 ×10^3^ and 1.22 × 10^4^ m ^−1^, respectively, in the low‐concentration range. The nonlinear plots versus the typical linear ones may be attributed to the presence of simultaneous static and dynamic quenching.^18^ The well‐documented electron transfer process from the conduction band (CB) of MOFs to the lowest unoccupied molecular orbital (LUMO) of the analyte, synergize with the interactions between hydroxyl group of TNP and free Lewis‐base sites (triazine core in **L**) in the fluorophore via electrostatic interactions to enhance the TNP quenching efficiency.^19^ Most likely, the guest TNP molecules are more orderly aligned and vibrate weakly at low temperature, which benefits the ligand and TNP interactions, improves the short‐range electron transfer, and enhances the quenching efficiency. Using **EuL** MOF to detect highly explosive TNP at room and even low temperatures is innovative, and expands the applicability for this efficient, economic, and portable fluorescent sensor.

## 3 Conclusion

In summary, an unprecedented fast, reversible capture and release of intrinsic Eu^3+^ in the porous **EuL** MOF via a SCSC transformation was observed. The transformation simultaneously involves the cleavage and formation of dynamic Eu–O bonds, ligand conformations change, and the tremendous symmetry change of MOF structures. At low temperature, the Eu^3+^ motion quickly decelerates, and it is fully captured by the spare carboxyl groups, which results in a structural deformation. Gradually increasing the temperature to room temperature causes the captured Eu^3+^ to escape the hooks and regains the structure. This interesting phenomenon represents a rare dynamic control between noncovalent and covalent interactions, and provides a designed synthetic route for new MOF materials. Our ongoing work will focus on exploring stimuli‐responsive structural transformations and switchable transformation‐dependent functionalities.

## 4 Experimental Section

*Synthesis of **1a***: A mixture of EuCl_3_·6H_2_O (0.0183 g, 0.05 mmol), Na_6_ **L** (0.0187 g, 0.025 mmol), and DMF (2 mL) was placed in a beaker and stirred for 10 min. The pH value of the mixture was adjusted to 4.3–4.8 using HNO_3_ (1 m) and NaOH (1 m) under stirring. And then, it was transferred to a 10 mL Teflon‐lined reactor and heated at 65 °C for 3 days. After it was cooled to room temperature, colorless block crystals were collected by filtration, washed with DMF and EtOH in sequence, and dried in air with a yield of 63% based on EuCl_3_·6H_2_O.

*Synthesis of **1b***: When the single‐crystal of **1a** was kept at −80 °C within approximately 10 min, it changed to crystal **1b**.

*Crystal Data of **1a***: C_54_N_12_O_28_NaEu_2_, *M_r_* = 1591.57, monoclinic, *C*2/*c*, *a* = 24.3176(16) Å, *b* = 23.9869(18) Å, *c* = 31.166(2) Å, *β* = 104.7100(10)°, *V* = 17583(2) Å^3^, *Z* = 4, linear absorption coefficient: *μ* = 0.743 mm^−1^, radiation wavelength: *λ* = 0.71073 Å, temperature of measurement: *T* = 293(2) K, 2*θ*_max_ = 52.26°, reflections collected/independent: 77297/17433, *R*_int_ = 0.1169. The final *R*_1_ = 0.0871 (*I* > 2*σ(I)*), *wR*_2_ = 0.2382 (*I* > 2*σ(I)*), GOF = 0.954.

*Crystal Data of **1b***: C_69_N_17_O_33_NaEu_3_, *M_r_* = 2073.73, monoclinic, *P*2_1_/*n*, *a* = 25.723(3) Å, *b* = 21.974(2) Å, *c* = 31.243(3) Å, *β* = 107.0240(10)°, *V* = 16886(3) Å^3^, *Z* = 4, linear absorption coefficient: *μ* = 1.149 mm^−1^, radiation wavelength: *λ* = 0.71073 Å, temperature of measurement: *T* = 193(2) K, 2*θ*_max_ = 47.68°, reflections collected/independent: 65880/24930, *R*_int_ = 0.0863. The final *R*_1_ = 0.0660 (*I* > 2*σ(I)*), *wR*_2_ = 0.1723 (*I* > 2*σ(I)*), GOF = 0.977. CCDC 1014506 (**1a**) and 1014507 (**1b**) contain the supplementary crystallographic data for this paper. These data can be obtained free of charge from The Cambridge Crystallographic Data Centre via www.ccdc.cam.ac.uk/data_request/cif.

## Supporting information

As a service to our authors and readers, this journal provides supporting information supplied by the authors. Such materials are peer reviewed and may be re‐organized for online delivery, but are not copy‐edited or typeset. Technical support issues arising from supporting information (other than missing files) should be addressed to the authors.

Supplementary

Supplementary
