# Supplementary material for: A Temperature‐Responsive Smart Europium Metal‐Organic Framework Switch for Reversible Capture and Release of Intrinsic Eu3+ Ions
Source: Adv Sci (Weinh). 2015 Mar 10;2(4):1500012. doi: 10.1002/advs.201500012 (PMC5115357; doi:10.1002/advs.201500012)
Supplement: Supplementary file 1 — Supplementary [file ADVS-2-0d-s001.pdf]

Copyright WILEY-VCH Verlag GmbH & Co. KGaA, 69469 Weinheim, Germany, 2015.

Supporting Information

for *Adv. Sci.*, DOI: 10.1002/advs.201500012

A Temperature-Responsive Smart Europium Metal-Organic Framework Switch for Reversible Capture and Release of Intrinsic Eu^{3+} Ions

Min Zhu, Xue-Zhi Song, Shu-Yan Song,* Shu-Na Zhao, Xing Meng, Lan-Lan Wu, Cheng Wang, and Hong-Jie Zhang*

Submitted to WILEY-VCH

Copyright WILEY-VCH Verlag GmbH & Co. KGaA, 69469 Weinheim, Germany, 2014.

Supporting Information

A Temperature-Responsive Smart Europium Metal-Organic Framework Switch for Reversible Capture and Release of Intrinsic Eu^{3+} Ions

Min Zhu,[a,b] *Xue-Zhi Song,*[a,b] *Shu-Yan Song,**[a] *Shu-Na Zhao,*[a,b] *Xing Meng,*[a,b] *Lan-Lan Wu,*[a,b] *Cheng Wang*[a] *and Hong-Jie Zhang**[a]

[a]State Key Laboratory of Rare Earth Resource Utilization, Changchun Institute of Applied Chemistry, Chinese Academy of Sciences, 5625 Renmin Street, Changchun 130022, China
[b]University of Chinese Academy of Sciences, Beijing, 100049, China

This file includes:

Materials and Methods

Supplementary Text

Figures S1 to S12

Tables S1 and S2

Scheme S1

Other Supplementary Materials for this manuscript include the following:

Video titled Transformation Mechanism

Table of Contents:

Text S1. Experimental Details.

1. Materials and general methods.
2. Synthesis of $(H_3O^+)Eu_{0.5}[EuNa_{0.5}$**L**$(DMF)(H_2O)]\cdot(solvent)_x$ (**1a**).
3. Photoluminescent sensing experiments.

Text S2. X-ray crystallography.

Table S1. Crystal data and structure refinements of compounds **1a** and **1b**.

Figure S1. Coordination geometries of Eu ions in compounds **1a** and **1b**.

Figure S2. View of the coordination geometry of Na ions and the Eu_2Na units.

Figure S3. The asymmetric units of **1a** and **1b**.

Figure S4. The overall three-dimensional structures of **1a** and **1b** view along the *a* and *b* axis.

Figure S5. The coordination modes of the ligand **L** in **1a** and **1b**.

Figure S6. Schematic view of the topologies for **1a** and **1b**.

Scheme S1. The fast and reversible SCSC transformation process between room temperature (RT) and low temperature (LT).

Figure S7. The TG analysis of **1a**.

Figure S8. The IR spectra of **1a** and Na_6**L**.

Figure S9. The simulated and the as-synthesized powder XRD patterns.

Figure S10. The solid-state excitation and emission spectra of **EuL** MOF at room temperature.

Figure S11. The luminescent spectra of **EuL** MOF with the temperature decreased from 300 K to 10 K.

Figure S12. The $^5D_0$ decay curves of **EuL** at 10 K and 300 K with emission monitored at 617 nm.

Table S2. The fluorescence lifetime with the temperature decreased from 300 K to 10 K.

Figure S13. View of the quenching percentage by different nitro-compounds.

Figure S14. The quenching effects on TNP at room temperature with gradually increasing concentration of TNP.

References

Text S1. Experimental Details.

1. Materials and general methods.

All commercially available chemical materials were of analytical grade and were used as received without further purification. The ligand 5,5’,5’’-(1,3,5-triazine-2,4,6-triyltriimino)tris-isophthalate hexasodium (Na_6**L**) was synthesized according to the previous literature.[S1]

IR spectra were recorded within the 4000-400 cm^{-1} wavenumber range using a Bruker TENSOR 27 Fourier Transform Infrared Spectrometer (FT-IR) with the KBr pellet technique and operating in the transmittance mode. Inductively coupled plasma-optical emission spectroscopy (ICP-OES) measurement was performed on a Thermo Scientific iCAP 6000 spectrometer. Thermogravimetric analysis (TGA) was performed on a Netzsch STA 449F3 TG/DTA instrument under air atmosphere. The samples were heated from room temperature to 800 °C with a heating rate of 10 °C/min. Differential scanning calorimetry (DSC) was performed on a Mettler Toledo DSC instrument from -100 °C to 25 °C with a heating rate of 10 °C/min. C, H and N microanalysis of **EuL** compounds was performed on a vario EL cube CHNS elemental analyzer. The room temperature experimental powder X-ray diffraction (PXRD) data at room temperature were collected on a Bruker D8-FOCUS diffractometer equipped with Cu Kα1 (λ = 1.5406 Å; 1600 W, 40 kV, 40 mA) with the step of 0.02°. The simulated PXRD patterns were calculated by using single-crystal X-ray diffraction data and processed by the free *Mercury v1.4* program provided by the Cambridge Crystallographic Data Center. The temperature-dependent luminescent properties were measured with a EDINBURGH FLSP-920 spectrometer equipped with a 450 W xenon lamp as the excitation source for steady measurment and a pulse xenon lamp (30 μs, 30 Hz) for time-resolved measurment.

2. Synthesis of $(H_3O^+)Eu_{0.5}[EuNa_{0.5}$**L**$(DMF)(H_2O)]\cdot(solvent)_x$ (**1a**).

A mixture of $EuCl_3\cdot 6H_2O$ (0.0183 g, 0.05 mmol), Na_6**L** (0.0187 g, 0.025 mmol) and DMF (2 mL) was placed in a beaker and stirred for 10 min. The pH value of the mixture was adjusted to 4.3-4.8 using HNO_3 (1 M) and NaOH (1 M) under stirring. And then, it was transferred to a 10 mL Teflon-lined reactor and heated at 65 ºC for 3 days. After it was cooled to room temperature, colorless block crystals were collected by filtration, washed with DMF and EtOH in sequence, and dried in air with a yield of 63% based on $EuCl_3\cdot 6H_2O$. Elem. anal. of **EuL** compounds, Found: C, 39.26; H, 3.87; N, 11.59%.

3. Photoluminescent sensing experiments.

The fluorescence emission spectra upon excitation at 345 nm were recorded with a Hitachi F-4500 spectrophotometer equipped with a 150 W Xenon lamp as an excitation source. The photomultiplier tube (PMT) voltage was 700 V, the scan speed was 1200 nm/min. The slit widths of excitation and emission were set the same all over sensing experiments.

(1) Quenching percentage determination:

At room temperature:

The fine grinding sample of **EuL** (1 mg) was immersed in THF (2 mL), treated by ultrasonication for 30 min to form the stable emulsion. The emission spectra of the emulsions before the addition of nitro-compounds were recorded. And then, the emission spectra were recorded again after that identical quantities (280 μL, 1 mM) of different nitro-compounds were added to the above emulsions.

(2) Stern-Volmer plots determination (Fluorescence quenching titrations)

At room temperature:

In typical experimental setup, the fine grinding sample of **EuL** (1 mg) was dispersed in 2 mL THF to form the stable emulsion, which was then added to quartz cuvette. The fluorescence upon excitation at 345 nm was measured in-situ after incremental addition of freshly prepared TNP solutions (1 mM). The emulsion was stirred at constant rate during experiment to maintain homogeneity. Additionally, the fluorescence measurement was carried out under regular intervals after analyte was added.

At low temperature:

In addition to the above experimental procedures, the quartz cuvette was placed in a mixture of liquid nitrogen and acetone (-94.6 ºC). After the emulsion with TNP was stirred at given regular interval, the quartz cuvette was taken out and without any delay mounted to the sample holder of the fluorescence spectrophotometer and the fluorescence spectrum was recorded.

The formula used to calculate the final concentration of analyte in the cuvette was listed as followed:

$$c(\text{analyte}) = \frac{c_0 \times V(\text{added})}{V(\text{added}) + V(\text{original})}$$

c(analyte): the final concentration of analyte in the quartz cuvette; c_0: the initial concentration of analyte solution; V(added): the volume of the analyte; V(original) = 2000 μL.

Caution! 2,4,6-trinitrophenol (TNP) is potentially explosive and should be handled with the necessary precautions.

Text S2: X-ray crystallography.

The X-ray intensity data for the two compounds (**1a** and **1b**) were collected on a Bruker SMART APEX-II CCD diffractometer with graphite monochromatized Mo-Kα radiation (λ = 0.71073 Å) operating at 1.5 kW (50 kV, 30 mA) at 293 K and 193 K, respectively. Data integration and reduction were processed with SAINT software.[S2] Multiscan absorption corrections were applied with the SADABS program.[S3] Both structures were solved by direct methods and refined employing full-matrix least squares techniques based on F^2 using the SHELXTL-97 crystallographic software package.[S4] All non-hydrogen atoms were refined with anisotropic temperature parameters except the coordination DMF molecule, aqua ligands and lattice solvent molecules in the two compounds. The disordered C and O atoms of the carboxyl groups in **1a** and coordinated water molecule in **1b** were refined isotropically using the atoms split over two or three sites with equal occupancy. Because guest solvent molecules of the two compounds were seriously disordered, it was impossible to refine by using conventional models appropriately. The contribution of the electron density associated with disordered solvent molecules was removed by the SQUEEZE subroutine in PLATON.[S5] All hydrogen atoms attached to carbon and nitrogen atoms were not generated and not taken into the molecular formula consideration. "ISOR" commands were used to solve the NPD and ADP problems arising from the poor quality of the diffraction data. In compound **1b**, in order to rationalize the geometries of coordinated DMF molecules, "DFIX" comment was used to refine the related atoms. The detailed crystallographic data and structure refinement parameters for these compounds are summarized in Table S1.

Table S1 Crystal data and structure refinements of compounds **1a** and **1b**.

	**1a** (squeezed)	**1b** (squeezed)
Empirical formula	$C_{54}N_{12}O_{28}NaEu_2$	$C_{69}N_{17}O_{33}NaEu_3$
Formula weight	1591.57	2073.73
T, K	293(2)	193(2)
Crystal system	Monoclinic	Monoclinic
Space group	$C2/c$	$P2_1/n$
a/Å	24.3176(16)	25.723(3)
b/Å	23.9869(18)	21.974(2)
c/Å	31.166(2)	31.243(3)
α/deg	90	90

β/deg	104.7100(10)	107.0240(10)
γ/deg	90	90
V/Å^3	17583(2)	16886(3)
Z	4	4
F(000)	3076	3988
reflections collected	77297	65880
independent reflections	17433	24930
R_{int}	0.1169	0.0863
GOF on F^2	0.954	0.977
R_1[a], I > 2σ(I)	0.0871	0.0660
wR_2[b] I > 2σ(I)	0.2382	0.1723

[a] $R_1 = \Sigma||F_o|-|F_c||/\Sigma|F_o|$; [b] $wR_2 = \Sigma[w(F_o^2-F_c^2)^2]/\Sigma[w(F_o^2)^2]^{1/2}$

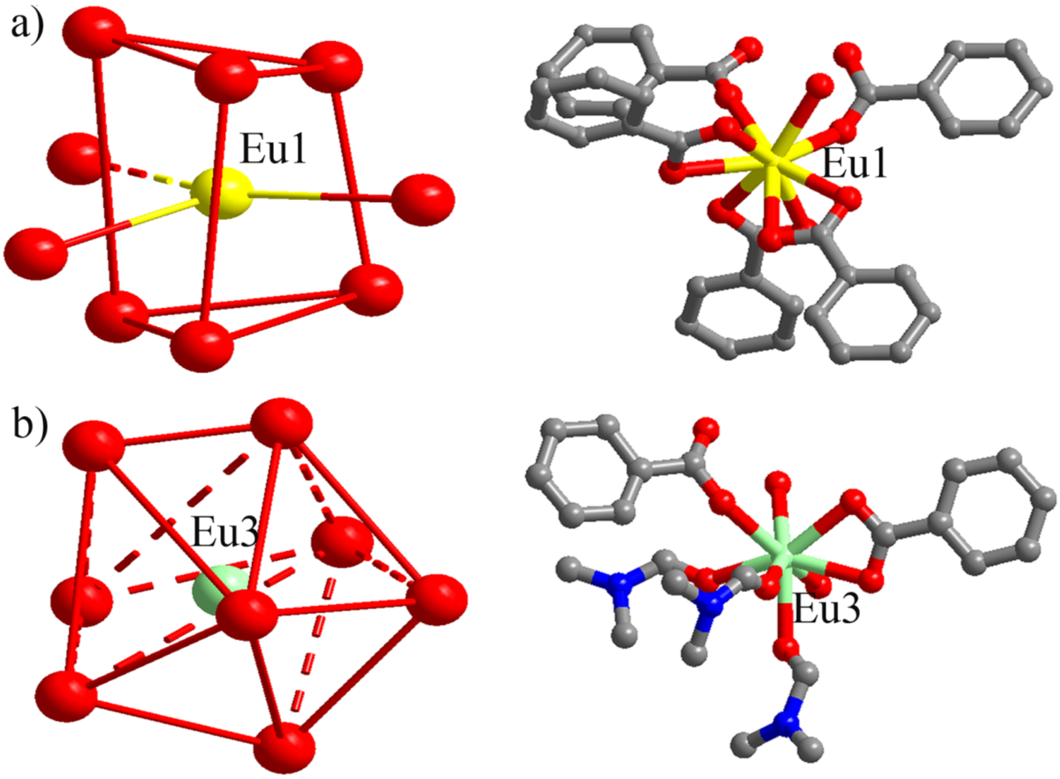

Figure S1. Coordiation geometries of Eu1 ions in compounds **1a** and **1b** (a), and Eu3 ions in compound **1b** (b).

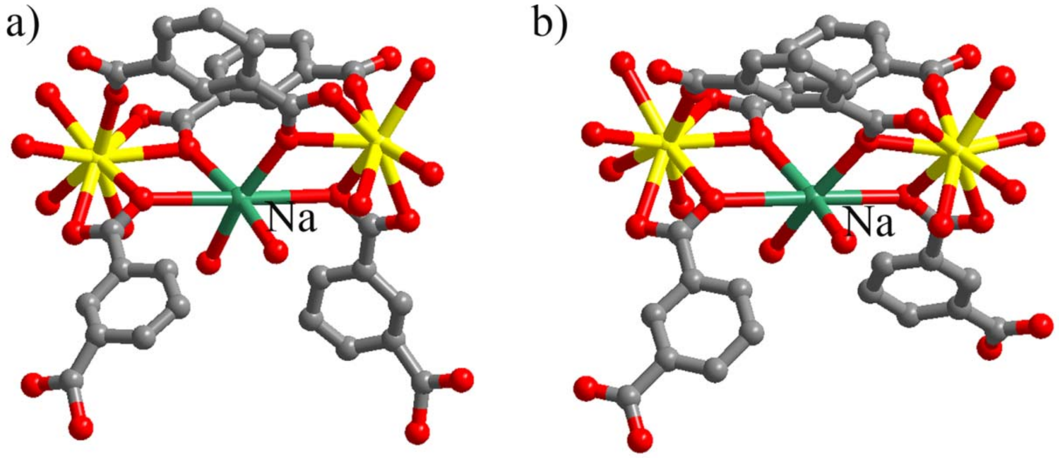

Figure S2. View of the similar coordination geometry of Na ions and the Eu_2Na units in **1a** (a) and **1b** (b).

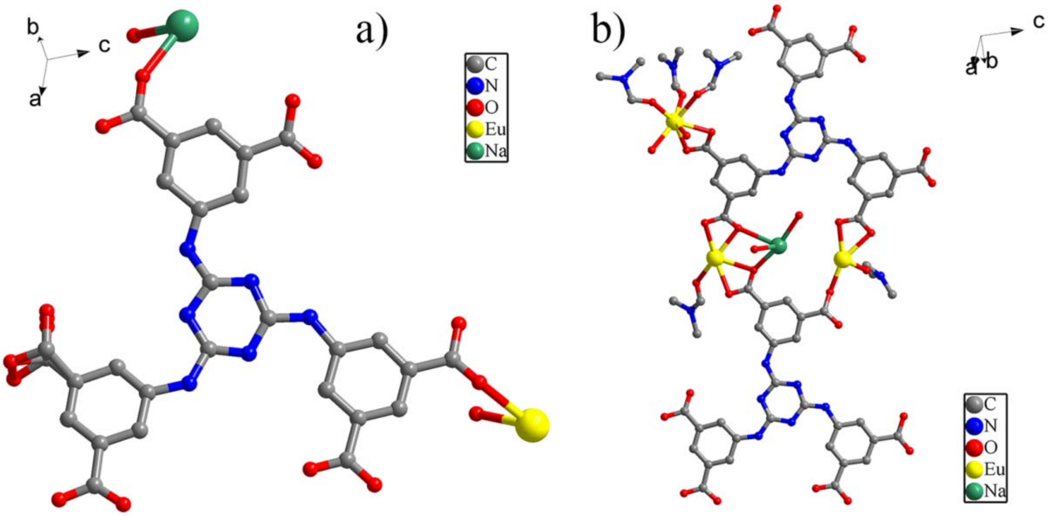

Figure S3. The asymmetric units of **1a** (a) and **1b** (b).

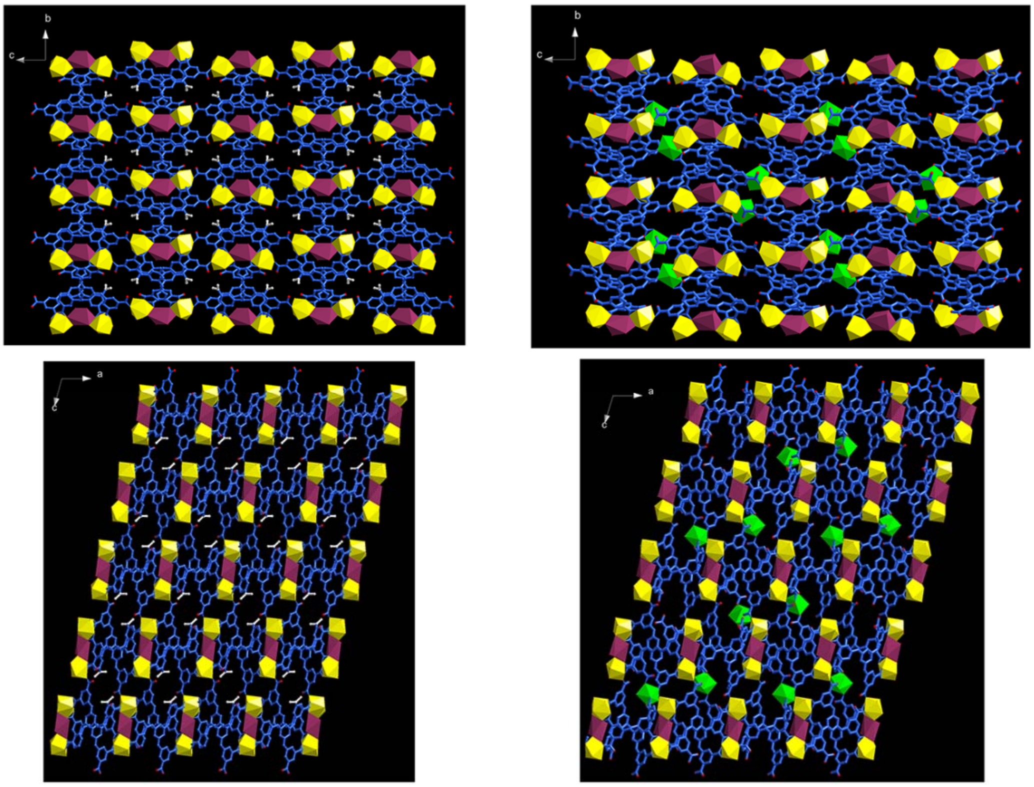

Figure S4. The overall three-dimensional structures of **1a** (left) and **1b** (right) view along the *a* (Top) and *b* (Bottom) axis.

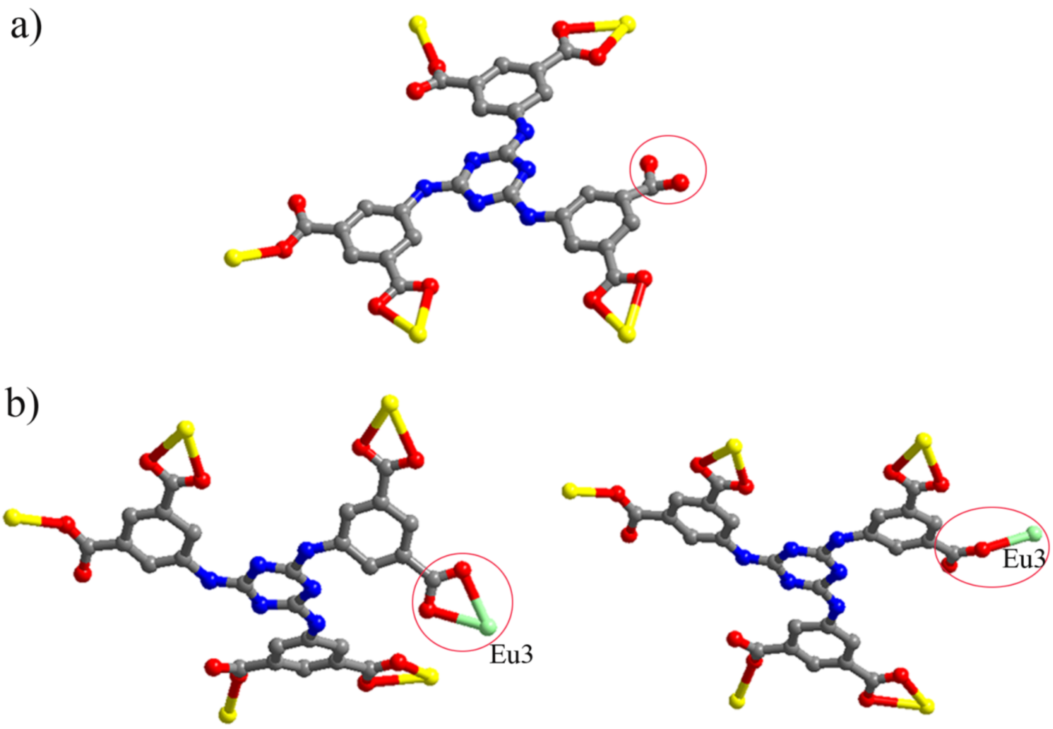

Figure S5. The coordination modes of the ligand **L** in **1a** (a) and **1b** (b).

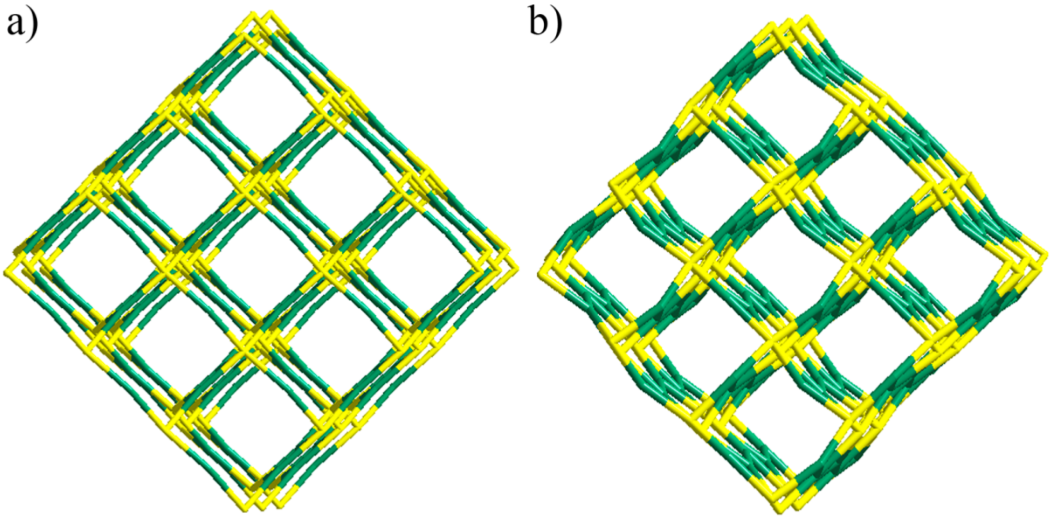

Figure S6. Schematic view of the binodal (3,6)-connected framework in **1a** (a) and the binodal (4,6)-connected framework in **1b** (b).

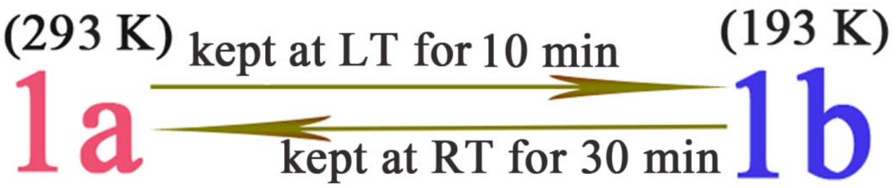

Scheme S1. The fast and reversible SCSC transformation process between room temperature (RT) and low temperature (LT).

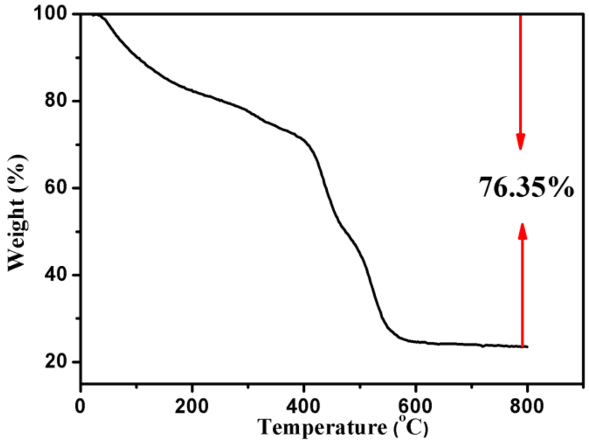

Figure S7. The TG analysis of **1a**.

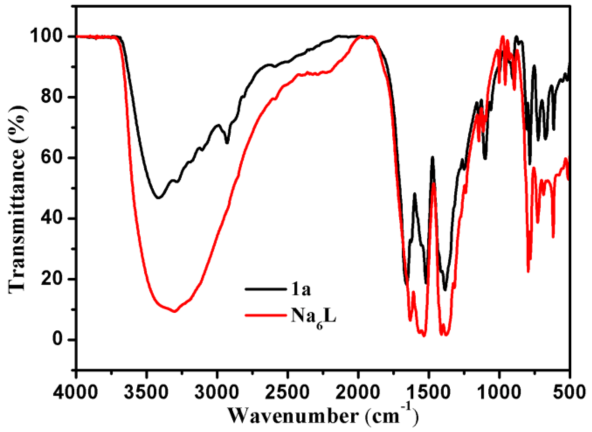

Figure S8. The IR spectra of **1a** and Na_6**L**.

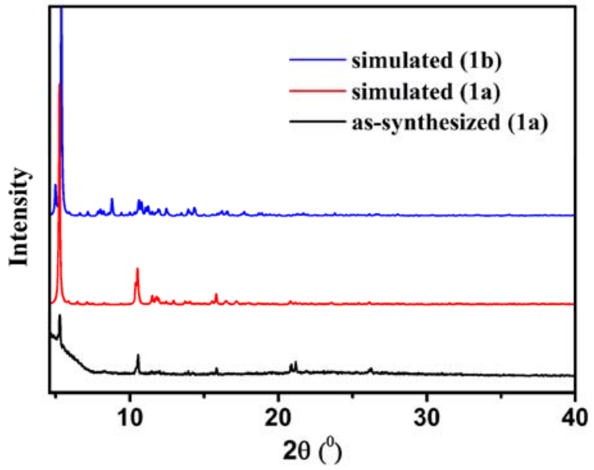

Figure S9. The as-synthesized XRD patterns of **1a** and the simulated powder XRD patterns of **1a** and **1b**.

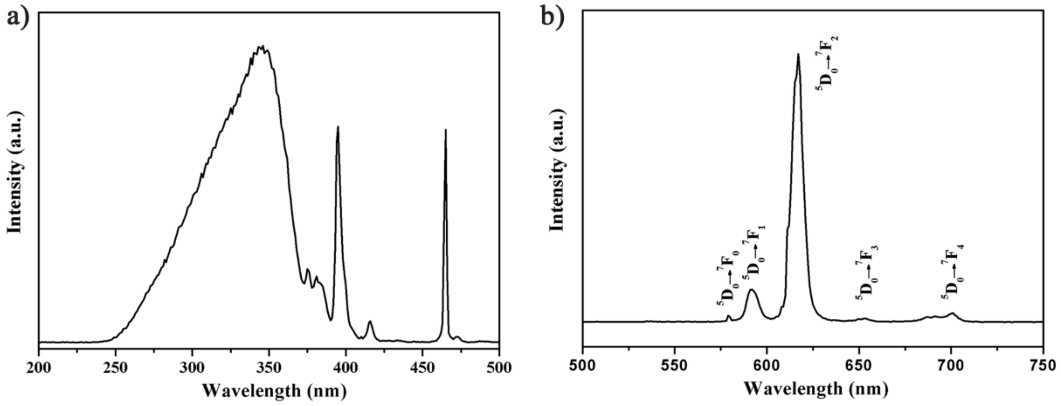

Figure S10. a) The excitation spectrum of **EuL** MOF monitored at 617 nm and b) emission spectrum excited at 345 nm at room temperature.

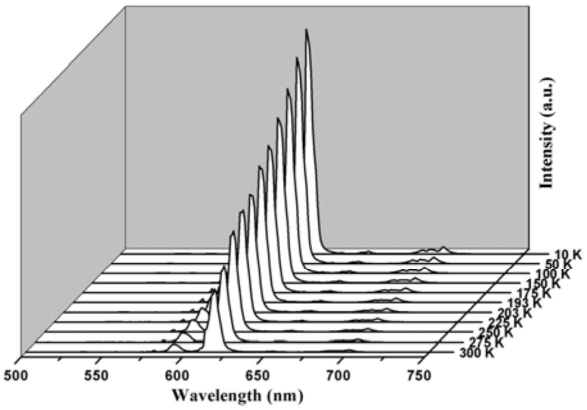

Figure S11. The luminescent spectra of **EuL** MOF with the temperature decreased from 300 K to 10 K.

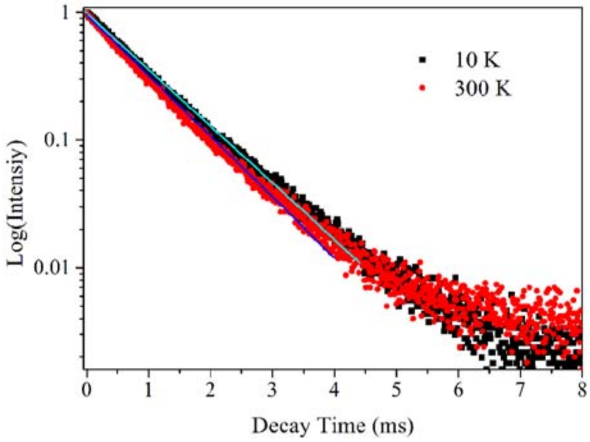

Figure S12. The $^5D_0$ decay curves of **EuL** at 10 K and 300 K with emission monitored at 617 nm.

Table S2 The fluorescence lifetime with the temperature decreased from 300 K to 10 K.

Temperature (K)	Lifetime τ (ms)
300	0.8294
275	0.8459
250	0.8627
225	0.8700
203	0.8784
193	0.8947
175	0.9001
150	0.9054
100	0.9154

50	0.9236
10	0.9267

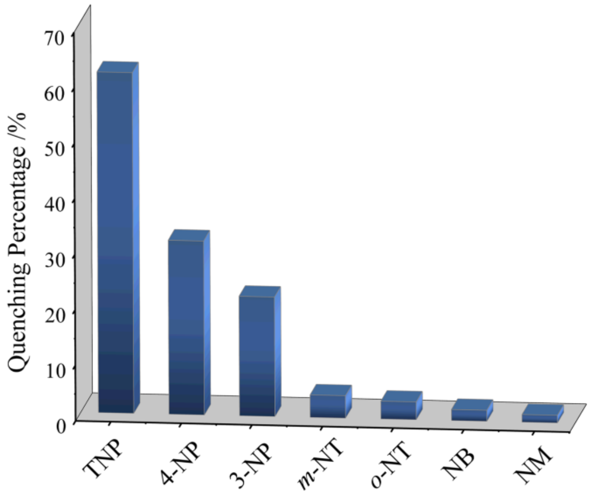

Figure S13. View of the quenching percentage by different nitro-compounds.

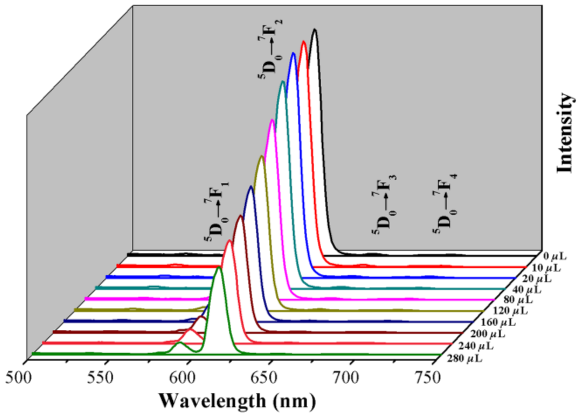

Figure S14. The quenching effects on TNP at room temperature with gradually increasing concentration of TNP.

References

[S1] R. Luebke, J. F. Eubank, A. J. Cairns, Y. Belmabkhout, L. Wojtas, M. Eddaoudi, *Chem. Commun.* **2012**, *48*, 1455.

[S2] SAINT, Program for Data Extraction and Reduction, Bruker AXS, Inc., Madison, WI, **2001**.

[S3] G. M. Sheldrick, SADABS, University of Göttingen, Göttingen, Germany, **1996**.

[S4] a) G. M. Sheldrick, SHELXS 97, Program for the Solution of Crystal Structure, University of Göttingen, Göttingen, Germany, **1997**; b) G. M. Sheldrick, SHELXS 97, Program for the Crystal Structure Refinement, University of Göttingen, Göttingen, Germany, **1997**.

[S5] P. Van der Sluis and A. L. Spek, *Acta Crystallogr., Sect. A: Found. Crystallogr.* **1990**, *46*, 194.
